# The sleep and activity database for the early years (SADEY) study: design and methods

**DOI:** 10.1186/s44167-024-00054-8

**Published:** 2024-06-18

**Authors:** Dylan P. Cliff, Devan Antczak, Catherine E. Draper, Timothy Olds, Rute Santos, Diego Augusto Santos Silva, Mark S. Tremblay, Esther M.F. van Sluijs, Byron Kemp, Eivind Aadland, Katrine Aadland, Thayna Alves Bezerra, Jade Burley, Valerie Carson, Hayley E. Christian, Marieke De Craemer, Katherine Downing, Kylie D. Hesketh, Rachel A. Jones, Nicholas Kuzik, Reetta Lehto, Clarice Martins, Jorge Mota, Andrea Nathan, Anthony D. Okely, Eva Roos, Eduarda Sousa-Sá, Susana Vale, Sandra Wiebe, Ian Janssen

**Affiliations:** 1 School of Education, Early Start, Faculty of the Arts, Social Sciences and Humanities, University of Wollongong, Northfields Ave, Wollongong, Australia; 2 SAMRC Developmental Pathways for Health Research Unit, University of the Witwatersrand, Johannesburg, South Africa; 3 Alliance for Research in Exercise, Nutrition and Activity (ARENA), University of South Australia, Adelaide, Australia; 4 Research Centre in Child Studies, University of Minho, Braga, Portugal; 5 Institute of Education, University of Minho, Braga, Portugal; 6 Federal University of Santa Catarina. Florianopolis, Santa Catarina, 88040900 Brazil; 7 Healthy Active Living and Obesity Research Group, Children’s Hospital of Eastern Ontario Research Institute, Ottawa, Canada; 8 MRC Epidemiology Unit, University of Cambridge, Cambridge, UK; 9 Department of Sport, Food and Natural Sciences, Faculty of Education, Arts and Sports, Western Norway University of Applied Sciences, Campus Sogndal, Sogndal, Norway; 10 Department of Physical Education, Regional University of Cariri, Crato, Ceará Brazil; 11 Faculty of Kinesiology, Sport, and Recreation, University of Alberta, Edmonton, Canada; 12 Telethon Kids Institute, The University of Western Australia, Perth, Australia; 13 Department of Rehabilitation Sciences, Faculty of Medicine and Health Sciences, Ghent University, Ghent, Belgium; 14 Institute for Physical Activity and Nutrition (IPAN), School of Exercise and Nutrition Science, Deakin University, Geelong, VIC Australia; 15 Department of Pediatrics, University of Ottawa, Ottawa, ON Canada; 16 Folkhälsan Research Center, Program for Public Health, Helsinki, Finland; 17 Research Centre in Physical Activity, Health and Leisure (CIAFEL), Faculty of Sport, University of Porto, Porto, Portugal; 18 Laboratory for Integrative and Translational Research in Population Health (ITR), University of Porto, Porto, Portugal; 19 Politécnico do Porto, Escola Superior de Educação, Porto, Portugal; 20 School of Health and Society, Early Start, Faculty of the Arts, Social Sciences and Humanities, University of Wollongong, Northfields Ave, Wollongong, Australia; 21 CIDEFES (Research Center in Sport, Physical Education, Exercise and Health), Universidade Lusófona, Lisbon, Portugal; 22 Department of Psychology, University of Alberta, Edmonton, AB Canada; 23 School of Kinesiology and Health Studies, Department of Public Health Sciences, Queen’s University, Kingston, Canada

**Keywords:** Physical activity, Sedentary behaviour, Movement behaviours, Early childhood, Data pooling, Data harmonisation

## Abstract

**Background:**

Canada, Australia, the World Health Organization and other countries have released 24-hour movement guidelines for the early years which integrate physical activity, sedentary behaviour, and sleep, focusing on supporting children to achieve a healthy 24-hour day. The guideline evidence synthesis, however, highlighted the dearth of high-quality evidence, particularly from large-scale studies. The Sleep and Activity Database for the Early Years (SADEY) project aims to assemble a large, pooled database of 24-hour movement behaviours and health indicators in young children (birth to 5.99 years), to advance knowledge in these areas. This paper describes the SADEY design and methods.

**Methods:**

Data sets were identified with > 100 children and device-measured (hip-worn ActiGraph accelerometers) physical activity and sedentary behaviour, parent-reported or device-measured sleep, and at least one health outcome: physical (BMI, waist circumference, blood pressure), social-emotional (Strength and Difficulties Questionnaire), cognitive (Early Years Toolbox), or motor development (Test of Gross Motor Development 2). Led by the University of Wollongong co-ordinating centre, the SADEY project collates the datasets to create a pooled database.

**Findings:**

To date, 13 studies from 7 countries have been included in the database. Ethics clearance and data sharing agreements have been secured for all studies and the SADEY 1.0 database is being assembled including ~ 8,000 participants.

**Discussion:**

SADEY will be used to address questions of global importance to public health policy and practice, for example – *Is the mix of movement behaviours across the 24-hour day associated with healthy development?, What is the optimal mix of these behaviours?*, and; *What factors can be targeted to support young children in achieving the optimal mix of 24-hour movement behaviours?* Additionally, SADEY seeks to develop and disseminate protocols, develop capacity on the device-based measurement of movement behaviours, and seeks partnerships with stakeholders that promote knowledge translation on movement behaviours to support healthy development among young children.

## Background

The term “movement behaviours” conceptualises movement on an intensity continuum, including sleep, sedentary behaviour, and physical activity. Collectively these mutually exclusive behaviours comprise the full 24-hour day. Globally, policies and strategies aimed at promoting optimal health and development among young children through physical activity and sleep have traditionally targeted these behaviours in isolation [1]. A recent paradigm shift has occurred, focusing more holistically on supporting children to achieve a healthy 24-hour day in relation to movement and sleep [1]. Since 2017, Canada [2], Australia [3], New Zealand [4], South Africa [5], and the World Health Organization [6] have released 24-hour movement guidelines for the early years which integrate physical activity, sedentary behaviour, and sleep. The evidence synthesis process to develop the guidelines has, however, highlighted the dearth of quality evidence to inform them, particularly from large-scale studies (*n* > 500) with direct measures. Considerable research is in progress and has been conducted in this area, and members of the guideline development committees have identified medium- to large-scale international datasets (n ~ > 100) of device-measured physical activity and sedentary behaviour, and parent-reported or device-measured sleep in 0-5.99-year-olds that could potentially be harmonised and merged into a single database. The researchers leading these projects have expressed their initial interest in being involved in a collaborative initiative.

Wearable activity monitors are the method of choice for directly measuring physical activity and sedentary behaviour in young children because proxy subjective reports from adults are inherently inaccurate at estimating the duration and intensity of physical activity [7]. While most studies using direct measures are relatively small, pooling and harmonising data across studies is achievable where the same wearable activity monitor has been used. A successful example of data pooling from wearable monitors is the International Children’s Accelerometer Database (ICAD) [8–10]. ICAD is a consortium of 20 partners wherein data were centrally collated and re-processed to create harmonised accelerometer variables in over 37,000 young people aged 2 to 18 years across studies from Europe, the USA, Brazil and Australia. In addition, non-accelerometer data were harmonised using transparent procedures [8, 9]. ICAD has provided globally significant evidence, for example, to understand the consequences [11], determinants [12] and prevalence [10] of physical activity and sedentary behaviour in youth.

With respect to understanding early childhood movement behaviours, ICAD has the following limitations: (i) the database includes limited data on young children: only three studies from Europe including 1,044 2–4-year-olds and 2,879 5–6-year-olds: (ii) data were collected up until 2008, and (iii) these studies do not consistently have data on sleep and developmental outcomes. In collaboration with ICAD, our research team conceptualised SADEY. We held a meeting with interested researchers at the International Society of Behavioral Nutrition and Physical Activity (ISBNPA) Annual Meeting (June 2017) and discussed this initiative. With the support of interested researchers, we formed a Leadership Group to govern the project, with appropriate content and technical expertise, and broad geographical representation. We held a leadership meeting in Hong Kong (August 2017), where vision, mission and objectives of the project were developed. These were defined as follows:

### Vision

To advance knowledge on movement behaviours, their determinants and related health and developmental benefits for children of the early years.

### Mission

(1) To assemble a large, pooled database of device-measured physical activity and sedentary behaviour, device-measured or parent-reported sleep, and their determinants and related health and developmental benefits in children of the early years. (2) To inform and build capacity in the measurement, analysis, and reporting of these behaviours in this age group.

### Objectives

For children of the early years, we will:

1. Describe the types, intensities, durations, patterns, and quality of movement behaviours.
2. Determine the types, intensities, durations, patterns, quality, and combinations of movement behaviours associated with optimal health and development.
3. Determine the intrapersonal, interpersonal, and environmental correlates of movement behaviours.
4. Develop and disseminate protocols and develop capacity, particularly in low- and middle-income countries (LMICs), on how to directly measure, analyse, and report on movement behaviours.
5. Seek partnerships with stakeholders that promote knowledge translation on movement behaviours.

The purpose of this paper is to describe the approach and methods used to develop the SADEY 1.0 database.

## Methods

### Design

SADEY is a harmonised database of de-identified secondary data from cross-sectional and longitudinal studies on young children’s 24-hour movement behaviours (including device-measured physical activity and sedentary behaviour, and device-measured or reported sleep), health and developmental indicators, and sociodemographic and descriptive characteristics.

### Ethics approval

Ethics approval for the SADEY project was granted by the University of Wollongong’s Human Research and Ethics Committee (2021/249). Additionally, ethics approval was obtained for all contributing studies, in line with relevant local and national regulations. Data contributors were required to share this approval before contributing to SADEY.

### Roles

SADEY is an international collaborative project between the SADEY Leadership Group, SADEY Working Group, Data Contributors and Data Users. Led by the SADEY Leadership Group Co-Chairs (DC, IJ), the SADEY leadership group (TO, MT, EvS, DASS, CD, RS), with their expertise in children’s 24-hour movement behaviours, international collaborative and data-pooling studies, and evidence translation, provides oversight of the project. The SADEY Working Group (DA, DC, IJ), based at the University of Wollongong co-ordinating centre, oversees the daily operations and implements the project, drawing on the institution’s expertise in database management. Following the completion of Collaboration Agreements, Data Contributors become formal collaborators on the project. Data contributors obtain ethics approval to share de-identified data with the co-ordinating centre and are invited to contribute as co-authors to proposed studies that include their study data. Along with the SADEY Leadership Group, Data Contributors are given 12-months advanced access to the database and can apply with study proposals to use the data. Once the advanced access period has ended, the database will be accessible to external researchers (i.e., Data Users) who can propose studies using the data via the same process as Data Contributors. Details about accessing the database, including release dates and application forms, will be provided on the SADEY website (https://www.uow.edu.au/global-challenges/living-well-longer/early-years-accelerometry/).

### Recruitment of studies and datasets

To contribute to the database, studies needed to meet the following inclusion criteria: Design – Observational (cross-sectional or longitudinal) or experimental (baseline data for cross-sectional analyses and follow-up data for longitudinal analyses from the control group only); Participants – population-based samples of children aged birth to 5.99 years (at the first assessment for longitudinal or experimental studies); Measures – SADEY 1.0 is restricted to studies that used hip-worn ActiGraph accelerometers to measure children’s physical activity and sedentary behaviour, and that also have parent-reported or device-measured sleep (from the hip-worn ActiGraph accelerometer). The rationale for this restriction is that the ActiGraph is widely used in studies among young children and has established acceptability, validity, and reliability [7]. Studies must also have collected at least one of the following outcomes: Physical development (measured height and weight to calculate Body Mass Index [BMI], waist circumference, or blood pressure), social-emotional development (Strengths and Difficulties Questionnaire [13]), cognitive development (Early Years Toolbox [14]), or motor development (Test of Gross Motor Development [15]).

### Participants

To date, the database contains data from 13 studies representing seven countries (Australia, Belgium, Brazil, Canada, Norway, Finland, Portugal) and includes > 7,500 children with at least 1 day of valid accelerometry and reported sleep (defined as at least 6 h of valid daytime wear [16]; Table 1) and at least one outcome. Six of these studies are longitudinal. Eleven studies were conducted among 3- to 5.99-year-olds at baseline, while two studies involved toddlers (1-2.99 years-old).

**Table 1:** Studies included in the SADEY 1.0 database

Study name	Location	Age group (years)	Design	24-hr wear protocol	*N* with at least 1 valid day^a^ (N1)	Total measured days (N1)	*N* with at least 3 valid days^a^ (N3)	Total measured days (N3)	*N* with reported sleep	BMI	WC	Blood pressure	Executive function	Psychosocial health	Gross motor skills
ACTNOW [34]	Norway	3–6	Longitudinal	x	1227	7823	1193	7766	1129	x	x		x	x	x
DAGIS [35]	Finland	3–6	Cross sectional	x	850	5663	837	5643	806	x	x				
GET UP [36]	Australia	1–3	Longitudinal	x	292	1359	221	1260	224	x	x	x	x	x	
HAPPY [37]	Australia	3–5	Longitudinal		903	5206	809	5062	940	x					
Jump Start [38]	Australia	2–4	Longitudinal		433	2059	351	1932	0	x			x		x
PACE [39]	Canada	3–5	Cross sectional		92	583	90	580	97	x			x		
PATH-ABC [40]	Australia	3–5	Longitudinal		282	1716	274	1702	332	x	x	x	x	x	
PLAYCE [41]	Australia	2–5	Cross sectional		1746	9667	1528	9340	1558	x				x	
PREPS [42]	Canada	1–3	Cross sectional		185	1016	163	988	251	x					
Prestyle [43]	Portugal	3–5	Cross sectional		392	2291	368	2249	366	x	x	x			
PROXDEV [44]	Canada	3–5	Cross sectional	x	107	682	103	676	107	x			x		x
The Movement´s Cool Project [45]	Brazil	3–5	Cross sectional		236	1240	207	1196	267	x	x		x		x
TOYBOX [46]	Belgium	3–5	Longitudinal		953	4732	912	4662	919	x	x				
Total					7698	44,037	7056	43,056	6996						

^a^ = defined as a day with at least 6 valid accelerometer daytime wear hours, BMI = Body Mass Index, WC = Waist Circumference

### Measures

#### Movement behaviour data

**Physical activity and sedentary behaviour.** Physical activity and sedentary behaviour were measured in all contributing datasets using ActiGraph accelerometers (ActiGraph Corporation, Pensacola FL). Versions of ActiGraph monitors (including former versions called CSA/MTI) have been used in research studies to estimate physical activity among young children for more than two decades [17]. In a recent review of physical activity measurement tools used among young children [18], the ActiGraph was categorised as having “good” criterion validity against measured energy expenditure, “good” convergent validity at determining physical activity intensity and sedentary behaviour against direct observation, and “good feasibility”. The device provides time-stamped acceleration data that can be used to calculate time spent in physical activity or sedentary behaviour [7]. In each study, children wore the monitor around their waist on an elastic belt at the right hip, during waking hours (or in some cases for the full 24 h) for at least one day. Data reduction involves reprocessing original raw activity ActiGraph files (.gt3x), or count-based files of at most 15-second epochs (.agd) when raw files are unavailable, from each study. Processing involves the application of standardised, evidence-guided rules [7], with options for data users to apply their own decision rules (e.g., the number of hours of waking wear time to determine a valid day, and different cut-point definitions of sedentary behaviour and light, moderate or vigorous physical activity; see Harmonisation).

**Sleep.** Studies collected data on children’s typical sleep duration over a 24-hour period (i.e., including night-time sleep and day-time naps) using either parent-reports or hip-worn ActiGraph accelerometers. For studies with 24-hour accelerometer wear protocols, data reduction involves reprocessing ActiGraph raw or .agd files from each study using an algorithm validated in preschool-aged children [19] (See Harmonisation).

#### Developmental outcome data

All outcome measures have established, standardised/automated protocols which enable data harmonisation [9].

**Physical development.** Data on children’s measured height and weight will be used to calculate children’s BMI (weight (kg)/height (m)^2^), and age- and sex-specific BMI *z-*scores based on international definitions [20–22]. Some studies also collected data on children’s waist circumference, which will be analysed as a measure of central adiposity (including waist-to-height ratio). Likewise, several studies used a portable sphygmomanometer to collect systolic/diastolic blood pressure.

**Social-emotional development.** Several studies included data from the Strengths and Difficulties Questionnaire (SDQ) [13], which is one of the most widely used screening tools for mental health in young children internationally and provides an indication of young children’s social emotional development. The tool has established validity and reliability for use among young children, and the parent-report version has demonstrated acceptable internal consistency (Cronbach’s alpha = 0.70 to 0.80) and test-retest reliability (Intraclass correlation coefficient = 0.62 to 0.91) [13, 23, 24]. The SDQ includes 25 parent-reported items assessing scales of conduct problems, hyperactivity, emotional symptoms, peer problems and prosocial behaviour. Study outcomes include SDQ sub-scale (Emotional symptoms, Conduct problems, Hyperactivity, Peer problems, Internalising and Externalising problems, and Prosocial behaviour) and total scores.

**Cognitive development.** Several studies have measured cognitive and language development using the electronic tablet-based Early Years Toolbox (EYT) [14]. The measures include four tasks used to assess key executive functions; 1) visual-spatial working memory (‘Mr Ant’) and phonological working memory (‘Not This’) 2); inhibition (‘Go/No-Go’); and 3) task shifting (‘Card Sorting’). A fifth task assesses expressive vocabulary. Scores from these five tasks can be used in analyses. The EYT was psychometrically tested and normed among 1,764 preschool and early primary school students and results demonstrated that the tool displayed very good reliability (Cronbach’s α 0.84 to = 0.95), convergent validity with existing measures (*r* = .42 to 0.60), and developmental sensitivity (i.e., significantly higher scores with increasing age) [14].

**Motor development.** The Test of Gross Motor Development, 2nd Edition (TGMD-2) [15], was used in several included studies to assess children’s motor development. TGMD-2 is a process-oriented assessment (i.e., assesses the movement pattern rather than the outcome) that is widely used internationally and has been normed in a sample of 1,208 3 to 10 year-old children [15]. The test is comprised of six locomotor skills (run, gallop, hop, leap, horizontal jump, and slide) and six object-control skills (striking a stationary ball, catch, stationary kick, basketball dribble, overhand throw, and underhand roll). The tool has established validity for assessing young children’s fundamental movement skill proficiency as assessed through examination of the tool’s content validity, criterion-prediction validity and construct identification validity [15]. Reliability of the TGMD-2 has also been established through evaluation of the tool’s replicability in the areas of content sampling, time sampling and inter-scorer differences [15]. Test-retest reliability coefficients ranged from 0.86 to 0.91 for locomotor skills and from 0.71 to 0.94 for object control skills [15]. Data will include sub-test total scores, age and sex-adjusted locomotor and object control skill standard scores, and the gross motor quotient.

#### Sociodemographic variables and other behavioural data

In addition to the child’s age and sex, data pertaining to the family’s sociodemographic status is requested from Data Contributors. These data include the parent’s employment status, income level, marital status, and level of education. Not all studies included in the database have collected these variables in the same way. For example, in some studies the parent education variable may have three levels (i.e., less than high school, completed high school, and more than high school) while other studies may have five levels (i.e., no schooling, less than high school, completed high school, university degree, post-graduate degree). Therefore, several iterations of harmonised variables have been created (i.e., parent_education1, parent_education2, etc.). Lastly, the database includes variables on children’s screen use (e.g., average daily total screen duration) as reported by parents.

### Harmonisation

The SADEY Working Group initially drafted a data harmonisation protocol. The SADEY Leadership Group then reviewed and provided feedback on the proposed protocol. Through this iterative process, we reached consensus and finalised the SADEY harmonisation protocol.

Accelerometer data were harmonised by converting raw accelerometer files into counts per epoch applying the same procedures across all studies using R Statistical Software (v4.1.2 ) [25]. The R package ‘activityCounts’ has shown near perfect agreement (Cohen’s Kappa = 0.945) compared to Actilife, the proprietary Actigraph software, when converting raw accelerometer data to counts per second [26]. Data were then collapsed using the ‘PhysicalActivity’ package to 15-second and 60-second epochs for physical activity and sleep processing, respectively. To provide Data Users with different options to choose how they define sedentary behaviour, light-, moderate-, or vigorous-intensity physical activity in their studies, commonly used cutpoints developed by Pate et al. [27], Evenson et al. [28], and Trost et al. [29] were applied to the data separately to provide variables of the estimated time spent at different physical activity intensities (see Table 2). In addition to these established cutpoint methods, we report time spent in narrower cutpoint blocks within the intensity spectrum (referred to as “intensity spectrum blocks”) to provide greater flexibility within the range of physical activity intensities. The intensity spectrum blocks approach allows Data Users to choose the blocks that approximately match to different cutpoint thresholds (i.e., other than the Pate, Trost, or Evenson cutpoints) without requiring the data to be reprocessed (see Table 2). Likewise, average acceleration (counts per 15-seconds) is reported.

**Table 2:** Physical activity and sedentary behaviour cutpoints applied in SADEY accelerometer data reduction

Method	Cutpoints (counts/15 sec)
Pate [27]	SED = 0–37 L-LPA = 38-200 H-LPA = 201–419 MPA = 420–841 VPA = 842+
Evenson [28]	SED = 0–25 LPA = 26–573 MPA = 574–1003 VPA = 1004+
Trost [29]	SED = 0–48 LPA = 49–418 MVPA = 419+
Intensity Spectrum Blocks	0–13, 14–25, 26–63, 64–125, 126–188, 189–250, 251–375, 376–500, 501–625, 626–750, 751–875, 876–1000, 1001–1125, 1126–1250, 1251–1500, 1501–2000, 2000+

SED = sedentary time, L-LPA = lower light physical activity,

H-LPA = higher light physical activity, LPA = light physical activity,

MPA = moderate physical activity, VPA = vigorous physical activity,

MVPA = moderate-to-vigorous physical activity

Sleep periods were identified using the decision-tree algorithm in the PhyActBedRest package developed by Tracy et al. [19] which showed good sensitivity (0.936), specificity (0.970) and accuracy (0.952) compared to visual identification of sleep periods in a large (*n* = 400), socio-economically diverse sample of preschool-aged children (3 to 6 years). Additional sleep variables (e.g., sleep efficiency, wake after sleep onset, number of awakenings, average awakening duration, and sleep fragmentation index) were derived using the Sadeh algorithm [30]. Non-wear during waking hours was defined as ≥ 20 min of consecutive zero counts [31], while non-wear during sleep was defined as at least 90 min of consecutive zero counts with up to 2 min of non-zero interruption [32].

All accelerometer physical activity variables are reported for each day as well as for each hour of each day. This allows hour-by-hour analyses to be conducted to describe daily physical activity patterns and allows Data Users to apply their own decision criteria for defining a valid day based on the number of hours of valid data that they choose. Data users can also conduct sensitivity analyses with stricter accelerometer inclusion criteria to test if their results are impacted by such decisions.

Non-accelerometer data harmonisation largely followed steps outlined by the ICAD [9]. The harmonisation process involved reviewing the received data for consistencies and inconsistencies across studies to determine potential harmonisable variables. The process varied depending on the characteristics of the collected variables. For example, for a variable such as highest achieved parent education, studies offered different response options with different levels. To harmonise this variable across studies, multiple education variables were created with increasing levels of specificity. That is, at the simplest level, parent_education1 was coded with two levels: high school or less and more than high school. Additional education variables increased the levels and combinations of response options until all possible permutations were found. This will allow a data user who requests education for their analysis to use the most specific education variable that is common to all requested studies. A similar approach was taken with other variables (e.g., marital status and employment status). Outcomes that were measured with the same measurement tool were checked to make sure all the procedures were followed in the same way and variables reported in the same units (e.g., weight in kilograms and height in centimetres) and with the same variable coding. For example, data from the 25-item SDQ was received in different forms from each study and steps were taken to ensure that subscales were calculated in the same way across studies. Detailed documentation was recorded outlining the harmonisation procedures.

### Data sharing and storage

Once both the SADEY Working Group and Data Contributors have agreed to the terms of the SADEY Collaboration Agreement, and/or other data transfer agreement terms as requested by the Data Contributor, and the authorised representatives for each organisation have signed, then data sharing can begin. Data Contributors receive the SADEY Instructions for Data Contributors which outline the steps for de-identifying the data, formatting and preparing the data for transfer, and how to complete the transfer. Data Contributors share de-identified data via a secure cloud-based platform (AARNet FileSender). The received data are checked, processed and harmonised, and securely stored in a Structured Query Language (SQL) database using a virtual machine hosted by the University of Wollongong. The virtual machine allows for remote access to the database by Data Users to conduct analyses with appropriate ethics approval and an approved application to access the database.

### Data access

Potential Data Users complete the SADEY Application to Access Data Form, where they must describe the proposed research project, the data being requested, and must ensure that their project will use at least three contributing studies and not overlap with projects already in progress. Data available, ongoing or completed projects, and publications will be listed on the SADEY website (https://www.uow.edu.au/global-challenges/living-well-longer/early-years-accelerometry/). The SADEY Working Group reviews the application and determines whether (i) the research question is consistent with SADEY’s objectives in that it has a focus on early childhood movement behaviours (device-measured or parent-reported) and can be answered with SADEY data; (ii) there are obvious major flaws to the proposed research; (iii) the proposed research will require data from at least three studies that contribute to SADEY, to avoid duplication of findings and publications from original studies (i.e., re-analysis of a single study); and (iv) there is not a major overlap between the newly proposed project and a project already proposed by another researcher.

By completing the SADEY Application to Access Data Form, Data Users agree to the terms and conditions regarding data security and as outlined in the SADEY Publication and Authorship Policy which describes authorship and acknowledgment requirements from the SADEY Working Group, SADEY Leadership Group, and Data Contributors (see the SADEY website for forms - https://www.uow.edu.au/global-challenges/living-well-longer/early-years-accelerometry). Once an application is approved by the SADEY Working Group, Data Users are provided login details to the virtual machine and given access to only the variables in the database that they have requested in their application. Access is granted for 12 months. At the end of 12 months, Data Users are required to submit a SADEY Project Update Form where they can request an additional 12 months or indicate the project is complete. At present, the virtual machine is limited to the analytic software SPSS and R Studio; however, Data Users requiring other software applications can request them with their application form.

## Discussion

The SADEY 1.0 database represents a large pooled and harmonised international dataset on 24-hour movement behaviours during early childhood. The database provides an opportunity to address questions of global importance to public health policy and practice related to the prevalence, types, intensities, durations, patterns, correlates, and health and developmental benefits related to young children’s 24-hour movement behaviours. While many studies investigating movement behaviours in early childhood have been undertaken, only a very small number with device-based measures of physical activity and sedentary behaviour have been conducted among large samples (*n* > 500). With data from over 6,000 young children, SADEY provides a unique opportunity to conduct research into early childhood movement behaviours using a large sample. This will allow studies to test the strength and confidence in associations from smaller investigations on a larger scale and will provide the statistical power needed to examine associations for relatively small sub-samples (e.g., associations for obesity). Although other large-scale international studies involving primary data collection of preschool-aged children’s 24-hour movement behaviours are being conducted, such as the SUNRISE study [33], SADEY is positioned to make unique and significant contributions to the evidence base. SADEY includes longitudinal data, providing the opportunity to investigate later health benefits of early childhood 24-hour movement behaviours. The database also includes toddlers younger than 3-years of age, although this age group constitute a smaller sample. Additionally, SADEY will be accessible to researchers via an application process, providing the opportunity for new research teams with innovative ideas to collaborate on the project. The availability of data follows the policy of transparency and good research practices. Furthermore, there is potential for future iterations of the database to include additional datasets such as that from SUNRISE where data can be harmonised.

The database will initially be used to investigate questions about the associations between 24-hour movement behaviours and health and development among young children, for example ­­– *To what extent is the composition of movement behaviours across the 24-hour day associated with healthy development?, What is the optimal composition of these behaviours?*, and; *What factors can be targeted to support young children in achieving the optimal composition of 24-hour movement behaviours?* Because of the widespread adoption of 24-hour movement guidelines internationally, particularly by WHO [6], addressing these questions has important implications for global public health policy.

The initial stages of the project have focused on establishing the database. A limitation of the SADEY 1.0 database is that the included studies were predominantly conducted in high income countries. Future plans involve the inclusion of studies from low-to-middle income countries (LMIC) where data is available. Further, the SADEY project seeks to develop and disseminate protocols, and develop capacity, on how to assess movement behaviours with device-based measures and seeks partnerships with stakeholders that promote knowledge translation on movement behaviours. For example, SADEY has established a Memorandum of Understanding with the Active Healthy Kids Global Alliance, which is a not-for-profit organization made up of researchers, health professionals, and stakeholders who work together to advance physical activity in children and adolescents around the world. Likewise, relevant societies such as the International Society for Physical Activity and Health (ISPAH), and the International Society of Behavioral Nutrition and Physical Activity (ISBNPA) have established networks and capacity building initiatives. These have been identified as key organisations through which capacity building goals can be pursued, particularly to support research in LMIC. Such activities will allow the SADEY project to make important contributions to movement behaviour research and public health internationally.

## Data Availability

The data that support the SADEY database are available by application. Restrictions apply to the availability of the data in the database which are under licensed agreements. Further information about availability will be online at https://www.uow.edu.au/global-challenges/living-well-longer/early-years-accelerometry/.
